# Phenol in dermatology: updated evidence on efficacy and safety

**DOI:** 10.1016/j.abd.2025.501200

**Published:** 2025-08-25

**Authors:** Carolina Reato Marçon

**Affiliations:** Service of Dermatology, Santa Casa de São Paulo, São Paulo, SP, Brazil

**Keywords:** Chemical face peeling, Croton oil, Phenol, Rejuvenation, Substance-related disorders

## Abstract

Phenol, or carbolic acid, is an organic compound with caustic and antiseptic properties widely used in dermatology. Since its introduction as an antiseptic in the 19^th^ century, its use has expanded to various areas of medicine, including the treatment of dermatological conditions such as vitiligo, warts, guttate leukoderma, hidradenitis suppurativa, angiosarcoma, acne scars, alopecia areata, onychocryptosis, and actinic keratoses. In deep peels, phenol stands out for its effectiveness in skin rejuvenation, promoting intense and sustained neocollagenesis, with unparalleled results. Its ability to alter dermal structure makes it an essential therapeutic tool in various dermatological approaches. However, its use requires extreme caution due to its rapid cutaneous absorption and unique toxicokinetic profile. The substance can induce serious complications, such as cardiac arrhythmias, renal failure, neurotoxicity, and multiple organ failure, especially when applied to large areas or with inadequate techniques and formulations. Historical and contemporary studies report cases of fatal poisoning due to cutaneous exposure to phenol, highlighting the need for strict precautions in its use. To minimize these risks, it is essential that procedures be performed by highly trained physicians, with constant monitoring and controlled application, to ensure safety and maximize the therapeutic benefits of this substance, whose efficacy is widely recognized.

## Introduction

Phenol, or carbolic acid, is an organic compound derived from benzene, known for its caustic and antiseptic properties. Discovered in 1834 by Friedrich Ferdinand Runge through its isolation from coal tar, its clinical use began to gain prominence in the 19^th^ century, when British surgeon Joseph Lister introduced it as an antiseptic in 1865. Based on Louis Pasteur's discoveries about the germ theory of disease, Lister applied phenol to clean wounds and surgical instruments, revolutionizing medical practice by substantially reducing postoperative infection rates.^1^

**Figure 1:**
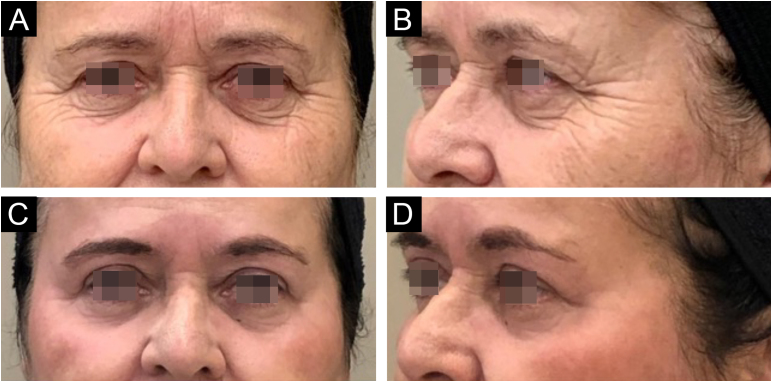
59-year-old patient, before (A and B) and after (C and D) 6 months of phenol-croton 35%/1.2% peeling for rejuvenation of the periocular region.

**Figure 2:**
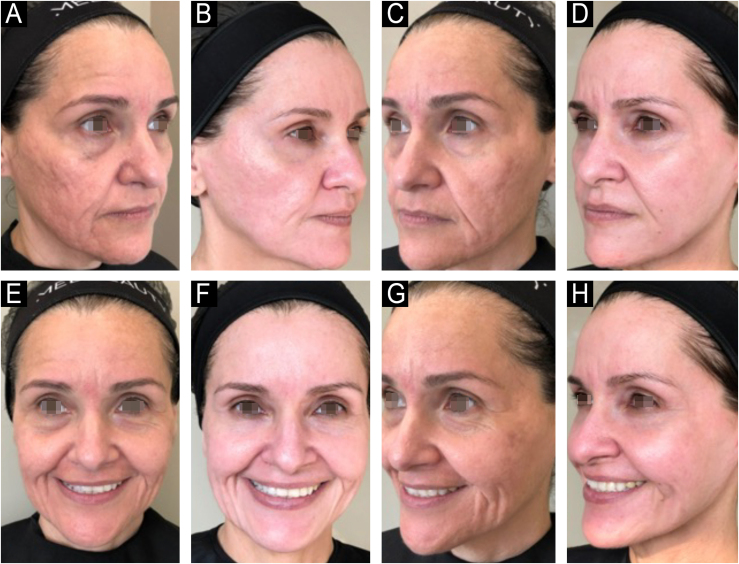
50-year-old patient, before and after one year: A and B (right hemiface); C and D (left hemiface); E and F (frontal face); G and H (right hemiface smiling), phenol-croton peeling 35%/1.6% for the treatment of acne scars and photoaging.

Since its introduction into medicine, phenol has established itself as a versatile therapeutic agent with wide-ranging applications, especially in dermatology. In deep peels, it stands out for its effectiveness in skin rejuvenation and the treatment of acne scars,^2^, ^3^ offering significant clinical results (Figure 1, Figure 2). Furthermore, phenol has therapeutic indications in a variety of dermatological conditions, including vitiligo,^4^ guttate leukoderma,^5^ common warts,^6^ melasma,^7^ actinic cheilitis,^8^ actinic keratoses, and Bowen's disease.^9^ With the advancement of medical practices, its use has expanded to other areas, such as in the treatment of onychocryptosis, where it acts as a sclerotizing agent, promoting the selective destruction of the nail matrix,^10^ in the management of pilonidal cysts^11^ and minor dermatological surgical procedures.^12^ Phenol has also demonstrated efficacy in inflammatory and proliferative conditions, such as hidradenitis suppurativa^13^ and keloids.^14^ The extent of its applications, combined with consistent therapeutic results, makes phenol a valuable resource in dermatology, meeting a diversity of clinical demands with high efficacy.

Despite the remarkable results achieved with phenol, both in aesthetic treatments and in other areas of dermatology, its use carries significant risks. Phenol systemic toxicity can lead to serious complications, such as cardiac arrhythmias and renal failure.^15^, ^16^, ^17^ Therefore, it is imperative that procedures involving this substance be performed exclusively by trained physicians and in controlled environments.

The objective of this review is to explore the main indications for phenol in dermatology, critically analyzing its therapeutic applications, efficacy, and potential risks. Furthermore, it seeks to emphasize the importance of a careful and safe approach to the use of this substance, highlighting the need for technical training and rigorous monitoring to ensure clinical efficacy and patient safety.

## Mechanism of Action, Toxicokinetics, and Metabolism

### Mechanism of Action

Phenol is a flammable and highly corrosive organic acid that is readily absorbed and widely distributed through all routes of exposure, including inhalation, cutaneous, and oral exposure. The substance has hydrophilic and lipophilic properties, which facilitate its penetration through cell membranes. Phenols denature and precipitate cell proteins, resulting in coagulation necrosis.^17^

**Figure 3:**
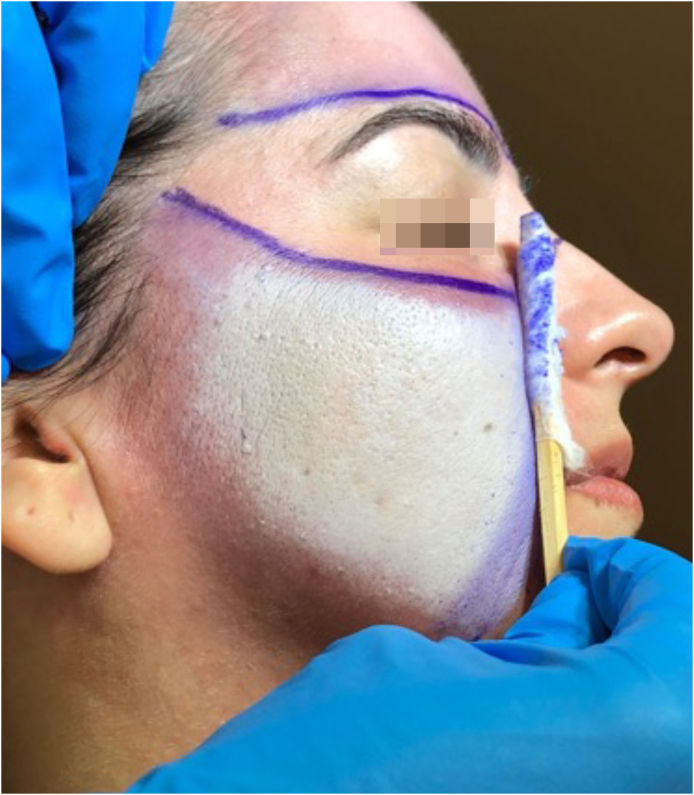
Uniform frosting that sets in quickly after applying phenol to the skin, resulting from the denaturation and coagulation of the proteins in the epidermal keratin and the superficial layers of the dermis.

On the skin, the substance has an immediate caustic effect, promoting the denaturation and coagulation of epidermal keratin proteins and the superficial layers of the dermis, clinically translated as rapid, uniform whitening (Fig. 3). Its caustic effect results in deep dermal remodeling, stimulating the production of collagen and elastin. This mechanism is widely explored in aesthetic procedures, such as facial rejuvenation, due to its ability to promote structural skin renewal with long-lasting results.^18^

### Absorption and Toxicokinetics

Phenol absorption into the bloodstream after cutaneous application is intense and rapid, with urinary recovery of 70% within 30 minutes and 85%–95% within 24 hours.^17^, ^19^ Even small amounts can reach significant serum levels, making strict dose control and the use of targeted application techniques essential.^16^

Studies indicate that cutaneous absorption is little influenced by the concentration of the applied solution but depends largely on the area of exposed skin.^20^ In 1971, Piotrowski studied solutions containing phenol at concentrations of 5, 10, and 25 mg/m^3^ and found that, regardless of concentration, the compound is rapidly absorbed through both the skin and the lungs.^21^

### Metabolism and Elimination

When applied to the skin, phenol is rapidly absorbed due to its lipophilic nature and low molecular weight, penetrating the stratum corneum and reaching the systemic circulation. Once absorbed, it undergoes hepatic detoxification, where approximately 75% is metabolized primarily by glucuronidation, sulfonation, and oxidation via CYP2E1. Elimination occurs predominantly through the kidneys, being excreted in the urine mainly in the form of glucuronides and sulfates. Approximately 25% of the phenol undergoes further metabolism, being converted to carbon dioxide (CO_2_) and water, while a small fraction may be excreted unchanged in the urine. Furthermore, in the first few minutes after absorption, some of the phenol may be eliminated through the lungs, which may be associated with the characteristic odor exhaled by patients. A smaller fraction of the phenol is eliminated in the feces, resulting from the biliary excretion of metabolites, although this pathway plays a secondary role in the total elimination of the compound. The half-life of phenol varies depending on the dose and route of exposure, but its efficient renal elimination generally prevents accumulation in healthy individuals. Therefore, adequate hydration and stimulating diuresis are interesting strategies to optimize its elimination. Furthermore, because respiratory, urinary, and biliary excretion occur relatively quickly, it is essential to maintain adequate intervals between successive applications in cosmetic units, ensuring complete elimination before further exposure.^19^, ^20^

## Dermatological Applications of Phenol

### Vitiligo

Studies report the use of 88% phenol for depigmentation of residual skin in advanced cases of vitiligo.^4^, ^22^ When applied topically in high concentrations, the substance has melanotoxic action, causing chemical necrosis of melanocytes. This toxic action on melanocytes compromises melanin synthesis in the treated areas, leading to permanent depigmentation and color uniformity. This effect is particularly useful in patients with generalized vitiligo, in whom repigmentation is unfeasible or unsatisfactory.^4^

A study published in the *Anais Brasileiros de Dermatologia* in 2005 reported the case of a 62-year-old patient with generalized vitiligo and residual normal-colored areas on the neck who was treated with 88% phenol. After two applications, 45 days apart, the pigmented areas were completely eliminated, with no signs of repigmentation after 18 months.^22^

### Guttate Hypomelanosis

A study by Ravikiran et al.^5^ evaluated the use of 88% phenol in the treatment of idiopathic guttate hypomelanosis. The compound promoted controlled chemical necrosis in the hypopigmented areas, triggering a local regenerative response that led to progressive repigmentation of the lesions. Twenty patients with 139 macules were treated with two monthly applications of 88% phenol. After three months, 64% of the macules showed repigmentation, with 45% showing greater than 75% improvement.

### Vulgar Wart

A study compared the efficacy of cryotherapy with liquid nitrogen to 80% phenol in the treatment of hand warts. Sixty patients were randomly divided into two groups and treated weekly for up to six weeks. At the end, 70% of the patients treated with cryotherapy and 82.6% of those treated with phenol achieved complete healing, with no statistically significant difference between the methods.^6^

Another study compared the efficacy of curettage and electrocoagulation with the application of 80% phenol in the treatment of warts. The treatments were performed simultaneously on different lesions of the same patient, in 17 patients. After six weeks, the cure rate was significantly higher in the curettage and electrocoagulation group (100%) compared to the phenol group (65%). The study concluded that curettage and electrocoagulation were more effective, but the application of phenol may be a less invasive alternative for pain-sensitive patients.^23^

### Hidradenitis Suppurativa

A study evaluated the use of crystallized phenol in the treatment of sacral hidradenitis suppurativa (HS). Twenty-five patients were treated with phenol, with a mean follow-up of 36.8 months. The oozing openings were dilated with forceps after local anesthesia, the hairs were removed, and phenol was applied. The cure rate was achieved in 60% of cases, with recurrence in 40%, most of which resolved with reapplications. The treatment showed a significant reduction in Hurley severity scores and Physician's Global Assessment of HS after therapy, with no reported side effects. Crystallized phenol has proven to be an effective, minimally invasive option with little impact on patients' daily lives.^13^

A recently published pilot study (2024) evaluated the efficacy and safety of phenolization in the treatment of hidradenitis suppurativa (HS). After local anesthesia and individualization of the fistulas, 88% phenol was applied (three passes with a soaked cotton swab) to 22 fistulas in 12 patients, resulting in significant improvement in more than half of the cases after one month, with healing within two weeks. The technique proved particularly useful in anatomical areas that are difficult to manage surgically and in small, non-draining fistulas. Tolerance was good, with mild and transient side effects.^24^

### Pilonidal Cyst

Phenol has been used in the treatment of pilonidal cysts due to its sclerotizing and antimicrobial properties. The application aims to destroy the cyst epithelium, promoting obliteration of the tract and reducing disease recurrence. Numerous studies indicate that the use of phenol, in crystallized or 80% liquid form, is an effective alternative for the treatment of pilonidal cysts.^25^

Dogru et al.^25^ evaluated the impact of crystallized phenol on the outpatient treatment of pilonidal disease in 41 patients. The substance was applied 107 times, with most patients (70%) requiring two to three applications. The average recovery time was 42.7 days, with a 95% success rate, and only two recurrences were observed after five and eight months.

A literature review on the use of phenol as a minimally invasive method for the treatment of pilonidal disease revealed a mean time to return to work of 2.3 days and a healing time of 20 days. The overall success rate was 87%, with a mean follow-up of two years. The most common complications were abscess and cellulitis, with a mean incidence of 8.9%.^26^

A meta-analysis published (2024) compared phenolization with surgical excision in the treatment of pilonidal cysts. The analysis included 14 studies, including five randomized controlled trials and nine non-randomized trials. Phenol treatment showed fewer complications, shorter surgical time (mean 22.76 minutes), faster return to daily activities (mean 10.11 days), and shorter healing time (mean 17.11 days) compared to surgical treatment.^27^

### Mucocele

Sacchidanand et al.^12^ evaluated the use of 88% phenol in the treatment of mucous cysts. Four patients were treated with intralesional phenol injection, without the need for local anesthesia. After application, ulceration of the lesions was observed, followed by complete healing within two weeks. The absence of intraoperative or postoperative bleeding, minimal surgical defects, and discreet healing offered an advantage to phenol over surgery.^12^

### Premalignant Lesions and Cutaneous Neoplasms

A prospective study evaluated the efficacy and prognostic relevance of 100% phenol peels in patients with actinic keratoses (AK) and Bowen's disease (BD) using clinical, histological, and immunohistochemical criteria. Forty-six patients (32 with AK and 14 with BD), aged 31 to 91 years, were included. Applications were performed monthly for up to eight sessions, and patients were monitored for at least one year after treatment. There was a complete response in 84.8% of cases. Success rates correlated with factors such as tumor thickness and expression of molecular markers (PCNA and cyclin A).^9^

A recent study (2024) compared the efficacy of phenol-croton peeling (Hetter 35%/1.6%) versus imiquimod 5% in the treatment of actinic cheilitis. Thirty-six patients were randomized into two groups: one received imiquimod 5% three times a week for 30 days, and the other received a single phenol-croton peeling session. At 56 days, 94% of patients treated with phenol-croton showed complete clearing of the lesions, compared to none in the imiquimod group (p < 0.01). Histological and clinical improvements were more significant in the phenol-croton group. Despite more intense initial adverse effects, the peeling proved to be superior in efficacy and recovery.^8^

A study by Kaminaka et al.^28^ evaluated the use of 100% phenol in the treatment of cutaneous angiosarcoma (AS) in three cases. After treatment, biopsies revealed severe degeneration of the tumor and endothelial cells in the dermis, with positive staining using the TUNEL (Terminal Deoxynucleotidyl Transferase dUTP Nick-End Labeling) method, a technique that detects DNA fragmentation associated with apoptosis. Phenol peeling demonstrated advantages such as simplicity, speed, low cost, manageable pain, and sustained efficacy, suggesting its use as a complementary therapy for AS.

### Alopecia Areata

Studies have explored the use of phenol as a therapeutic option for alopecia areata. The proposed mechanism of action involves stimulating hair follicles through growth factors and cytokines released in response to necrosis and inflammation. Furthermore, it is suggested that phenol may act directly on the germinal centers of follicles, promoting hair growth.^29^, ^30^

A study evaluated the efficacy of 88% phenol in 50 patients with stable alopecia areata, applying it every three weeks. Approximately 78% of patients showed a good to excellent response. There was a significant improvement in hair texture and pigmentation within nine weeks, with a progressive increase in hair density.^29^

A report by Kar and Singh^30^ described a 13-year-old patient with diffuse alopecia areata involving more than 50% of the scalp who was treated with five biweekly sessions of 88% phenol and four biweekly pulses of intravenous dexamethasone. The phenol induced controlled inflammation and hair regeneration, while the dexamethasone modulated immunity. After 4 months, there was significant terminal hair growth, with no recurrence within 6 months.^30^

### Keloid

Mseddi et al.^14^ investigated the use of 40% phenol in the treatment of keloids in 25 patients. The mean age of participants was 37.7 years, with a predominance of phototype IV (64%) and lesions located on the trunk (48%). After an average of 14.2 sessions, a 75.5% reduction in lesion size was observed, with 72% of patients reporting satisfaction with the treatment and 81% adhering to the protocol. There were no recurrences during the average follow-up of 12.8 months. The authors highlighted that the induction of controlled tissue necrosis promoted scar remodeling, improving skin texture and color.^14^

### Dark Circles

Platsidaki et al.^31^ evaluated the combination of 10% phenol and 20% trichloroacetic acid in the treatment of dark circles in 31 women. The peeling was applied with a cotton swab in three layers to the upper and lower eyelids until a homogeneous frosting endpoint was reached. The treatment showed significant improvement, especially in younger patients. The authors suggested that the combination of the two substances offers complementary mechanisms of action, providing effective cosmetic benefits without the potential adverse effects associated with the use of higher concentrations of each compound alone.^31^

A retrospective study analyzed the efficacy and safety of deep chemical peels in the treatment of constitutional dark circles. Fifty-five procedures were performed on 52 patients, mostly women (92%), with a mean age of 46 years and Fitzpatrick phototypes III–IV (89%). In most cases, a phenol formula (60%–65%) combined with croton oil (0.6–0.7%) was used. The results showed that 89% of patients experienced clinical improvement greater than 50%, with an average duration of benefits of 24 months. Furthermore, 69% maintained satisfactory results until the last follow-up. Complications were rare, with only 4% of cases presenting persistent erythema, without the development of scars.^32^

### Melasma

**Figure 4:**
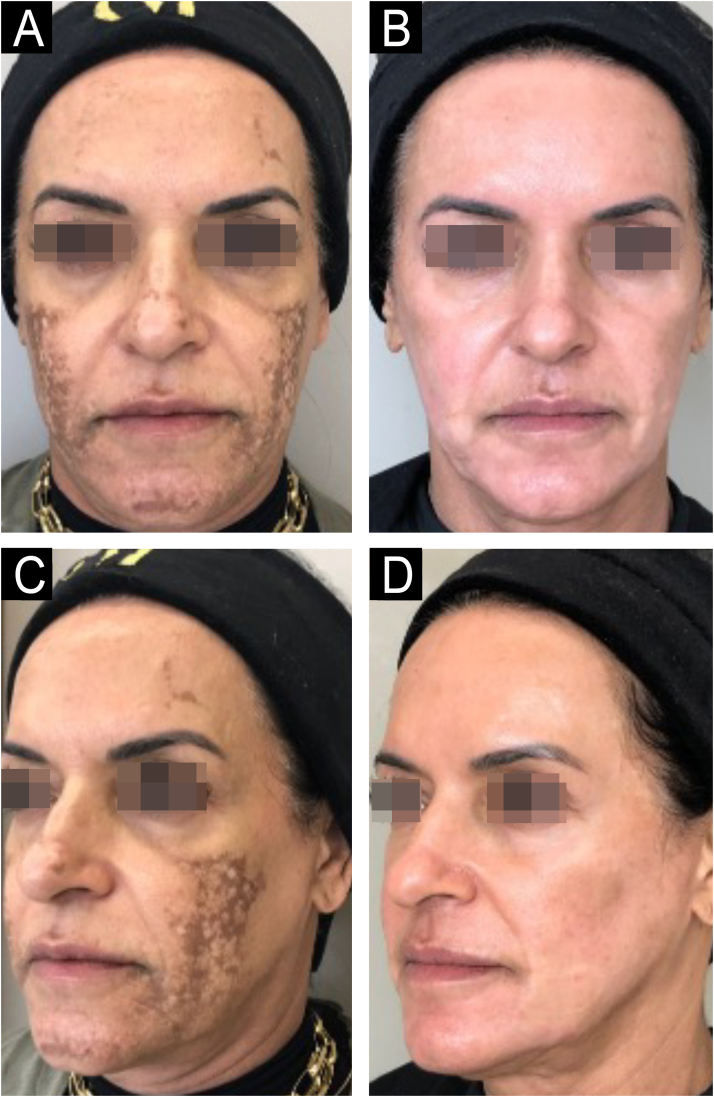
63-year-old patient with refractory melasma, before and after 6 months: A and B (frontal face); C and D (left hemiface), phenol-croton peeling 35%/1.2%.

A recent (2024) retrospective cohort study^7^ compared phenol peeling with moderate concentrations of Croton Oil (CO; 0.7–0.9%) versus higher concentrations (1.1–1.6%) in the treatment of recalcitrant melasma. Both groups showed significant improvement in mMASI after treatment and high scores on the Global Aesthetic Improvement Scale. However, the reduction was more pronounced in the group with CO < 1% compared to the group with CO > 1%. Remission was maintained for more than a year in 85% of cases, with no relevant differences between concentrations. The sustained improvement in melasma was attributed to the long-lasting treatment of elastosis, considered an important etiopathogenic substrate of melasma (Fig. 4). However, additional studies with larger sample sizes and longer follow-up are needed to confirm the stability of these results across different phototypes and clinical conditions.

### Onychocryptosis

Originally developed in 1945 by Boll,^33^ phenolization is one of the best-documented techniques in the medical literature for the treatment of onychocryptosis. Hundreds of studies, including extensive case series, demonstrate its safety and efficacy. Phenol acts as a sclerotizing agent, selectively destroying the nail matrix. Its benefits include a low recurrence rate, its antiseptic and analgesic properties, and the low risk of infection associated with the procedure.

A study published in 2024 analyzed the recurrence rate after partial matricectomy with 88% phenol, using a contact time of 45 seconds. A total of 1,460 surgeries were performed, with 802 patients followed for a six-month period. The recurrence rate was 0.75% at three months and 1.87% at six months.^10^

A retrospective study by Andreassi et al.^34^ analyzed 948 phenol cauterizations in 764 patients. There was no significant morbidity, and the procedure was well tolerated by all patients. The overall recurrence rate was 4.3% after 18 months. All recurrences were successfully treated with repeat application of the substance.

Other studies^35^, ^36^ corroborate these low recurrence rates, indicating consistency in the results. Furthermore, Cochrane reviews^37^, ^38^ and a recently published meta-analysis by Vinay et al. (2022)^39^ confirmed the efficacy and safety of phenolization, with minimal side effects.

### Acne Scars

**Figure 5:**
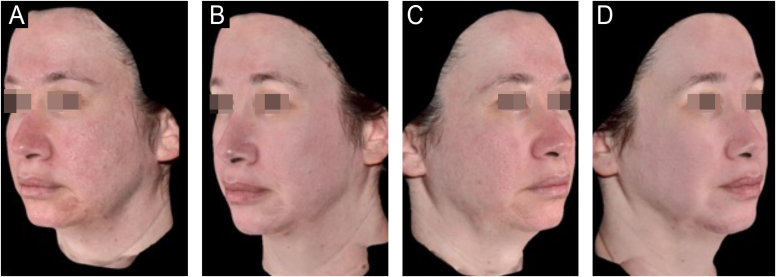
38-year-old patient with acne scars unresponsive to previous treatments (dermabrasion/CO2 laser), before and after 1 year: A and B (left hemiface); C and D (right hemiface), phenol-croton peeling 35%/1.2%.

The efficacy of phenol in the treatment of acne scars, with particularly notable benefits in deeper scars resistant to other procedures (Fig. 5), has been demonstrated in several studies. Mackee and Karp^40^ pioneered the use of phenol for acne scars, documenting its effectiveness in softening atrophic scars, a finding that supported the use of phenol decades later. Leheta et al.^41^ conducted a randomized clinical trial comparing a 60% phenol peel with croton (Skintech Inc., Spain) to Percutaneous Collagen Induction (PCI) combined with 20% Trichloroacetic Acid (TCA) treatment for post-acne atrophic scars. Twenty-four patients were divided into two groups, with Group 1 receiving one session of phenol peel and Group 2 four sessions of PCI with TCA. Both treatments resulted in significant improvement in scars, with the phenol peel showing slightly superior results, with an average improvement of 75.12% in Group 1 and 69.43% in Group 2.

Recent studies by Rullan and Rullan (2024)^3^, ^42^ explored the use of phenol in CROSS procedures (a technique of applying acids to individual scars) and in ring paint for boxcar and polymorphic scars. They observed that localized application of 89% phenol was effective, providing satisfactory results in improving scar texture and depth.

### Photoaging

**Figure 6:**
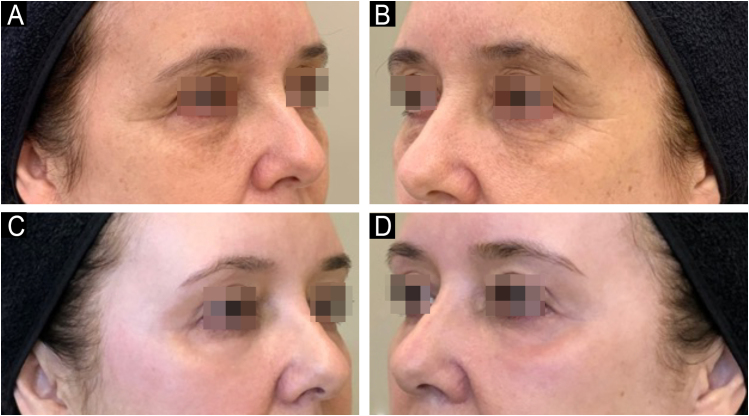
Rejuvenation of the periocular region with phenol-croton peeling 35%/1.2% in a 58-year-old patient. Before (A and B) and after (C and D) 6 months: A and C (right hemiface); B and D (left hemiface).

Several studies have analyzed the efficacy of phenol peels in the rejuvenation of photoaged skin, especially in combination with croton oil^2^, ^43^, ^44^ (Fig. 6). Butler et al.^45^ and Giese et al.^46^ showed that these peels significantly improve skin quality by stimulating the remodeling of elastic tissue. Hetter^47^ investigated phenol and croton oil formulas at various concentrations, highlighting the safety and efficacy of the procedure. Han et al.^48^ confirmed the rejuvenating effects in animal studies, while Kligman et al.^18^ performed histopathological analysis on skin biopsies from patients who had undergone peeling up to 20 years earlier, revealing the long-lasting effects of the procedure.

A recent study by Cardoso et al. (2022)^44^ evaluated 30 women with Glogau III–IV photoaging, showing that 87% achieved significant rejuvenation (Glogau I–II) three months after the peeling with 35% phenol and 1.2% croton oil. Another split-face study, conducted by Neitzke et al.^43^ compared the hemifaces of 48 patients. After six months, 79% showed rejuvenation of two or more points on an eight-point photonumeric scale, with a median improvement of three points (p < 0.0001). Another split-face study, which compared the classic Baker-Gordon peel (2.1% croton oil/50% phenol) and a modified Hetter formula (0.7% croton oil/33% phenol), showed similar efficacy, but with fewer side effects in the Hetter formula.^49^

### Other dermatological indications

Other therapeutic proposals include the treatment of oral leukoplakia, in which topical application of phenol prevented progression to malignancy,^50^ the treatment of hyperhidrosis, in which phenol is used for sympathectomy by chemical neurolysis,^51^ or as an alternative to the treatment of pyogenic granuloma.^52^

A literature review demonstrated that, outside the aesthetic context of deep peels, the use of phenol in other dermatological indications did not produce significant systemic adverse events. Reported complications were predominantly local, such as persistent erythema, burning sensation, scarring, and dyschromia. Phenol treatment has emerged as a simple, accessible, safe, and effective approach, with applications in a variety of dermatological conditions. Its use in small quantities reduces the risk of excessive systemic absorption and potential serious adverse effects, reinforcing its viability in well-conducted clinical practices.

## Phenol-Croton Peel

Before its formal adoption by physicians for cosmetic purposes, phenol-croton peeling was used empirically by laypeople in the early 20^th^ century. In the United States, aestheticians applied phenol solutions to remove wrinkles and facial blemishes, believing in its potential to “rejuvenate” the skin. However, this unregulated and unsupervised use often resulted in serious complications, infections, and permanent scarring.^53^

This scenario persisted for several decades, and the excellent but inconsistent results, in addition to the severe risks, caught the attention of physicians, who became interested in studying and controlling the use of the substance. Dr. Thomas Baker was one of the pioneers in formalizing the use of the substance in the aesthetic context. Along with his collaborator Howard Gordon, Baker dedicated himself to transforming the empirical use of phenol into a safe and scientifically based technique. The pair developed rigorous protocols to control peel depth and reduce complications, which resulted in the establishment of phenol peels as a medical procedure. The first scientific publication detailing the use of a combination of phenol and croton oil for cosmetic purposes appeared in 1961, laying the foundation for the understanding and refinement of the technique.^54^

Baker published his first formula with 47.5% phenol combined with 1.2% croton oil, adjusting it the following year to 2.1% croton oil.^54^, ^55^ In the 2000s, Hetter introduced variations on Baker's original formula, proving that the main agent in deepening the peel was croton oil and proposing fixing the phenol concentration at 35% and reducing and titrating the croton oil concentrations, ranging from 0.4% to 1.6%, in order to control the intensity of the peel and improve the safety profile of the procedure.^47^

**Figure 7:**
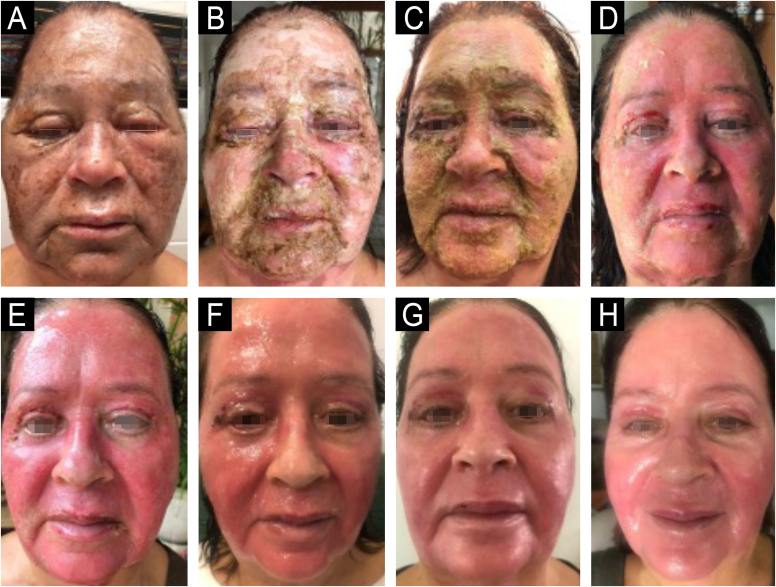
Day-by-day evolution (A-H), PO 1 to 8, from left to right horizontally, of the post-phenol-croton peel. Edema, intense neutrophilic inflammation, and removal of the outermost layers of the skin, replaced by rejuvenated and supple skin. Combined with phenol, croton oil enhances the penetration of the compound and acts synergistically on the production of collagen and elastin, providing more effective results. Post-procedure erythema may persist for up to 6 months.

Croton oil, extracted from the seeds of *Croton tiglium*, is rich in phorbol esters, which have intense pro-inflammatory activity, triggered by the activation of protein kinase C, which generates regenerative responses in the skin, stimulating neocollagenesis. The initial inflammatory response is triggered by the irritant activity of croton oil but is sustained and intensified by the pharmacological action of the plant's secondary metabolites (Fig. 7). These compounds, such as phorbol 12-myristate 13-acetate, induce the formation of a subcoagulative neutrophil band, assumed to be NETosis (programmed neutrophil cell death with release of extracellular DNA structures – Neutrophil Extracellular Traps). Phorbol esters also stimulate the secretion of cytokines by inflammatory cells in the dermis, promoting an increase in TGF-β (transforming growth factor beta) and, consequently, collagen production.^2^, ^56^ In combination with phenol, croton oil enhances the penetration of the compound, in addition to acting synergistically on the production of collagen and elastin, providing more effective results.^55^ Studies have shown that the combination of phenol with croton oil in the treatment of perioral wrinkles promoted an increase in the expression of SIRT-6 and SIRT-7 sirtuins, responsible for regulating collagen synthesis and protecting the skin against photodamage.^44^ Studies in animal models have shown a significant increase in type III collagen within 21 days after the procedure.^56^

Phenol-croton deep chemical peels are widely recognized as a leading technique in skin rejuvenation, offering long-lasting results unmatched by other methods. Robust studies support its efficacy, establishing it as an evidence-based recommendation for the treatment of photoaging. However, along with the evidence of effectiveness, concerns have arisen about the potential systemic risks associated with the procedure. Unlike other phenol applications, which have demonstrated safety under appropriate medical supervision, phenol-croton peeling is widely documented in the literature for its toxicity and potential risks.^57^, ^58^, ^59^, ^60^

## Systemic Risks and Evidence on Phenol Toxicity

### Overview of Systemic Toxicity

Records of phenol toxicity have been reported in the literature since the 19^th^ century. Numerous publications have highlighted the fatal potential of phenol formulations, whether through accidental or therapeutic exposure, via cutaneous, intravenous, oral, or inhalation routes. From the 1940s-61 to the present day, the authors have found publications that address the two antagonistic characteristics of the substance: the excellent effects of cutaneous applications and the serious risks resulting from systemic absorption and toxicity.

Early reports in animal models, such as those by Deichmann et al.^63^ point to the serious toxic effects of phenol when absorbed in large quantities, including everything from cardiac alterations to multiple organ failure. Historical reports, such as that by Lucas and Lane^64^ described cases of coma after dermal absorption of phenol. In 1922, Turtle and Dolan^65^ reported a fatal case in which a bottle containing phenol broke in a young man's pocket on his train ride home. Another case published by Miller in 1942^66^ reported an 18-year-old man with ringworm whose housekeeper applied a mixture of phenol and camphor to his shoulders and trunk, resulting in death within 15 minutes. In 2016, Giri et al.,^67^ published a report of four cases of acute phenol poisoning in children in India. The cases highlighted the multiple routes of absorption of the substance, as well as its complications in multiple organs. The severity of poisoning was more correlated with dermal exposure than with oral ingestion. The authors concluded that dermal exposure can rapidly progress to multiple organ failure, and that severity appeared to vary according to the degree of dermal exposure and the time interval to intervention.

Cases of dermal exposure to phenol resulting in lethargy and coma, nausea and vomiting, hypotension, cardiac arrhythmias, seizures, intravascular hemolysis, pulmonary edema, tracheal and bronchial inflammation, acidosis, methemoglobinemia, renal dysfunction, and coma have been reported. The reason for the predilection of some organs over others in different patients is unclear.^15^, ^68^, ^69^, ^70^

#### Systemic Risks

##### Central Nervous System

Neurological toxicity from phenol ranges from mild symptoms, such as lethargy, to seizures and coma. Phenol can exert a central narcotic effect, affecting the spinal cord and higher brain centers, producing rapid loss of consciousness and profound collapse.^71^

Griffiths^72^ reported a fatal case involving a 23-year-old man accidentally exposed to phenol, who died minutes after dermal contact, highlighting the risk of severe neurological toxicity. A 1900 publication by Abrahams^73^ described the case of a 7-day-old baby who was touched in the right groin by a nursemaid with phenol on her fingers. The child died 10 hours later, developing convulsions and a coma.

##### Cardiovascular System

Phenol can exert direct toxicity on the myocardium, causing cardiac arrhythmias, and on blood vessels, causing hypotension.^58^, ^74^ Studies in rats have shown decreased myocardial contraction and electrical activity after systemic exposure to the compound.^75^ The most common symptoms of phenol cardiotoxicity are tachycardia, bradycardia, bigeminy, and premature contractions, which can progress to ventricular tachycardia and atrial fibrillation.^58^, ^76^

Sex, age, previous cardiac history, and blood phenol levels were not accurate predictors of susceptibility to cardiac arrhythmia during phenol peeling.^76^, ^77^ Several authors agree^58^, ^61^, ^74^, ^78^^,^^79^ that absorption and cardiac toxicity are primarily influenced by the extent of skin exposed at a given time—and not by the concentration of the phenol solution—and recommend cardiac monitoring, slow and spaced applications, and forced diuresis as safety measures. However, some studies have shown that cardiac alterations were observed even with slow applications.^16^

A study by Truppman and Ellenby^76^ investigated the factors associated with the development of cardiac arrhythmias during facial phenol peels, focusing on variables such as procedure duration, size of the treated area, and formulation used. The study demonstrated that peels performed in less than 30 minutes, with 50% or more of the facial area treated, were highly correlated with the occurrence of arrhythmias. The saponified versus non-saponified formulation did not significantly influence the incidence of arrhythmic events, suggesting that the response is more dependent on the rate of phenol absorption than on individual characteristics or the formulation.^76^

Gross^77^ reported that 30% of 54 patients treated briefly with phenol peels developed some form of cardiac arrhythmia. Gross further noted that there were no predictable results regarding serum phenol levels and the occurrence of cardiac arrhythmia. However, there appeared to be suggestive evidence that segmented facial peeling, at intervals that allowed for metabolism and excretion of absorbed phenol, would reduce the risk.^77^

Beeson^80^ evaluated 43 consecutive patients who underwent deep peels with phenolic formulations. Ten developed cardiac arrhythmias, including premature ventricular contractions, bigeminy, paroxysmal atrial tachycardia, and ventricular tachycardia, highlighting the need for monitoring to identify and manage these potentially serious complications.^80^

A retrospective study evaluated 181 patients undergoing phenol peels. Patients were monitored multiparametrically throughout the procedure, received potent analgesics, conscious sedation, and intravenous hydration, and the room was adequately ventilated. Cardiac arrhythmia was recorded in 6.6% of patients, being more prevalent in those with diabetes mellitus, hypertension, and depression.^59^

Kadunc and Vanti^16^ investigated the systemic toxicity of phenol in facial peels, analyzing 60 women monitored via Holter monitoring and urinary gas chromatography (UGC), in addition to assessments of renal and hepatic function. The study revealed rapid elimination of phenol, with a half-life of 6 to 8 hours, and no significant changes in renal or hepatic function after 30 days. However, four patients experienced sustained ventricular tachycardia, highlighting the risk of cardiac complications. The authors concluded that, although phenol is effective, its use requires strict precautions and continuous monitoring to ensure patient safety.

A recent study by Rullan et al. (2024)^62^ evaluated the safety of phenol facial peels in 196 patients undergoing multiparametric monitoring during the procedure. Of the participants, ten (5%) experienced benign and transient arrhythmias: four had premature atrial contractions, two had premature ventricular contractions (one of whom also developed supraventricular tachycardia), and one had sinus tachycardia. One patient with initial sinus bradycardia experienced a temporary reduction in oxygen saturation, and three patients developed hypertension during the procedure. The results highlight the importance of intraoperative monitoring to mitigate cardiac and systemic risks associated with phenol use.^62^

Li et al.^81^ reported a fatal case of phenol toxicity in a 21-year-old man after attempting tattoo removal using an idiosyncratic method of subcutaneous injection of a “tattoo-eliminating solution” containing phenol at an unknown concentration. The session lasted three hours, after which he suddenly collapsed and died within minutes. There were no signs of underlying organic disease that could have contributed to the death. Toxicology revealed the presence of phenol in cardiac blood and liver tissue. Internal examination revealed pulmonary congestion and edema. Death was attributed to acute phenol-induced cardiotoxicity.

##### Urinary Tract

The mechanism by which phenol induces kidney damage is not clearly understood. Damage to renal tubular epithelial cells by free radical phenol intermediates, the inability of epithelial cells to form sufficient reduced glutathione to clear these intermediates, damage to the glomeruli by excretion of unconjugated phenol, renal ischemia, cast formation due to hemoglobin precipitation, extensive rhabdomyolysis, and/or hemoglobinuria due to intravascular hemolysis have all been proposed as possible mechanisms.^15^, ^19^, ^82^, ^83^ Seak et al.,^82^ Foxall et al.^83^ and Cohen et al.,^68^ have documented cases of renal failure in individuals with dermal exposure to phenol.

##### Respiratory System

Inhalation of phenol vapors can cause damage to the upper and lower respiratory tract. In more severe cases, inhalation of high concentrations can lead to glottis edema and acute pulmonary edema, serious and potentially fatal conditions. These effects are attributed to the corrosive nature of phenol, which damages the epithelial tissue of the airways and can induce an intense inflammatory response.^19^, ^84^

Although pulmonary toxicity through inhalation is most frequently discussed, dermal absorption of high concentrations can also cause respiratory impairment. The systemic distribution of phenol and its inflammatory action in target tissues, such as the lungs, is primarily responsible for these complications.^19^, ^21^, ^84^ Reports have documented cases of dermal and oral exposure to phenol associated with acute pulmonary edema and tracheobronchial inflammation.^68^, ^85^ Studies by Fu et al.^86^ and Gupta et al.^87^ described fibrosing alveolitis and Acute Respiratory Distress Syndrome (ARDS) after phenol exposure, reinforcing the severity of pulmonary impairment, particularly in the absence of adequate ventilatory support.

##### Liver

Phenol can induce acute hepatotoxicity due to the accumulation of toxic metabolites in the liver, which generate oxidative stress and mitochondrial dysfunction, leading to cell necrosis. Manifestations such as elevated liver enzymes, jaundice, and, in severe cases, liver failure may occur, requiring cautious use in therapeutic settings.^67^

##### Hematopoietic System

Cases of methemoglobinemia and intravascular hemolysis due to phenol exposure, in which oxygen transport was compromised, resulting in hypoxia and acidosis, have been described.^69^, ^88^ These are serious conditions that require immediate identification and intervention.

###### What is the safe dose of phenol for dermatological use?

Although the literature lacks precise data on safe doses for cutaneous absorption of phenol, several studies demonstrate that its toxicity is considerable at the concentrations and volumes commonly used in dermatological procedures such as phenol-croton peels. The toxicological literature offers a range for the Median Lethal Dose (LD50) of phenol in humans, which ranges from 50 to 500 mg/kg of body weight, resulting in an estimated dose of 3.5 to 35 g for a 70 kg person.^19^, ^84^

In validated preparations for deep peels, such as the Hetter formula, 10 mL contains approximately 3.5 g of phenol, while the Baker formula contains approximately 4.9 g of phenol in 10 mL, and an 88% phenol solution contains 8.8 g in 10 mL. Considering that 70% of topically applied phenol can be absorbed within 30 minutes, the authors would have an absorbed amount approaching lethal doses: 2.45 g, 3.4 g, and 6.6 g, in 10 mL, respectively.^19^, ^84^

The presence of ingredients such as croton oil and other additives common in irregularly marketed ready-to-use formulas, such as olive oil, cresol, sodium salicylate, camphor, glycerin, and soaps, among others, can potentially increase phenol's skin penetration, exacerbating the risk of systemic toxicity. The total volume of phenol, application methods, and dressing types are not standardized, posing an additional risk, especially when the procedure is performed by non-medical professionals.

###### Safety in Phenol Procedures

Safe handling of phenol requires rigorous protocols, including multiparameter monitoring, adequate room ventilation, and targeted application techniques. Recent studies, such as that by Rullan et al. (2024),^62^ indicate that complications are rare in settings with continuous monitoring and careful application. Other studies, such as that of Landau,^59^ demonstrate that factors such as pauses between applications and intravenous hydration are crucial to minimizing the risk of adverse events, especially in patients with comorbidities.

Toxicity is closely related to the excretion rate, area of application, and duration of application of the phenol solution.^79^, ^80^ Various organs in the body, including the heart, apparently can tolerate very low levels of phenol in the blood for short periods of time. It therefore seems essential to prevent phenol from being absorbed into the blood faster than natural processes can remove it.^16^, ^76^, ^79^

## Recommendations for the Safe Use of Phenol in Dermatology

### Pre-Procedure Evaluation

#### Rigorous and Holistic Screening

A complete medical evaluation of the patient prior to the procedure, including clinical and laboratory evaluations (renal and liver function, complete blood count, and complete metabolic profile), electrocardiography (24-h Holter monitoring), as well as analysis of current medications and skin diagnosis, is essential to minimize risks and ensure good results.^58^, ^59^, ^79^

Patients with a history of QT interval prolongation or use of medications that interfere with this interval are potentially more predisposed to more serious cardiac events.^57^, ^62^

#### Recommended Maximum Body Surface Area and Safety Criteria by Treated Area

**Figure 8:**
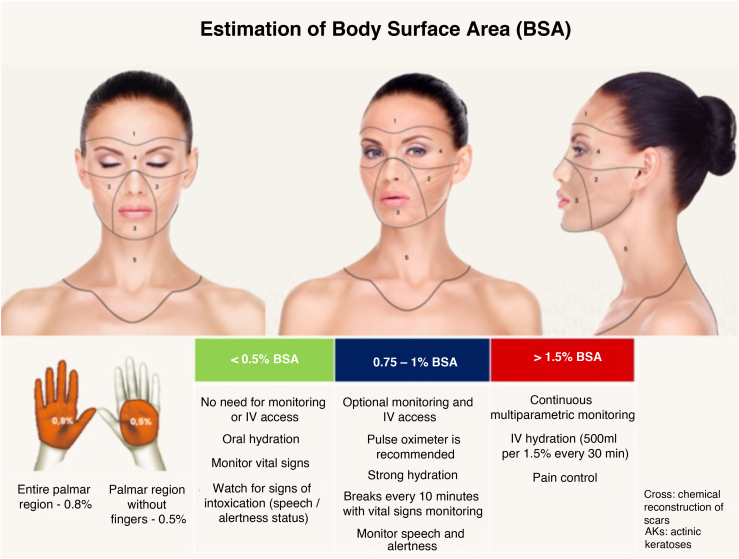
Division of the face into 5 cosmetic units for segmented and spaced application of the phenol-croton peel, ensuring procedure safety. Each unit represents 0.5%–1.5% of the Body Surface Area (BSA). The entire face plus the anterior neck represents 5% of the BSA. The perioral region with cheek feathering represents 1% of the BSA, and the periocular region (“Zorro Mask”) represents 1.5% of the BSA. In procedures with BSA greater than 1.5%, the patient must be monitored multiparametrically, safety breaks must be strictly enforced, and intravenous hydration must be instituted. Photos: Valua Vitaly. Editing and illustration: Ednei Ramos.

The risk of systemic toxicity from phenol is directly related to the amount of skin surface area treated in a given time period. It is recommended to limit the procedure to a maximum of 5% of the Body Surface Area (BSA), which corresponds approximately to the face and anterior neck (Fig. 8). In cases where the treated BSA exceeds 1.5%, multiparametric monitoring of the patient is essential throughout the procedure. Furthermore, safety pauses must be strictly enforced, and intravenous hydration must be instituted.^89^, ^90^, ^91^ Therefore, a practical categorization of dermatological indications is proposed according to the exposed area (Fig. 8).

1 Procedures with application to up to 0.5% of the BSA:

These include the treatment of conditions such as mucoceles, warts, guttate leukoderma, localized alopecia areata, onychocryptosis, small pilonidal cysts, and isolated scars.

In this context, systemic absorption is minimal, and there is no need for continuous monitoring or intravenous access. The following are sufficient: Oral hydration; Pain control with topical and/or oral analgesia; Clinical attention to alertness.

2 Procedures with application between 0.75% and 1.5% of BSA:

It covers medium-sized areas such as the upper third of the face, perioral region, or periorbital region, with feathering for the cheeks. These procedures require additional care: Intermittent monitoring and peripheral venous access; Intravenous hydration (optional); Safety breaks of 10 to 15 minutes after every 0.5% BSA; Strict control of the time and area treated; Continuous clinical attention.

3 Procedures with application to >1.5% of BSA (maximum 5%):

It includes facial peels (such as phenol-croton) in full-face or Zorro mask applications (Fig. 8) and extensive dermatoses. In these cases, mandatory safety measures include: Continuous multiparametric monitoring (ECG, BP, HR, SpO₂); Mandatory intravenous hydration; Safety breaks of 10 to 15 minutes per 0.5% ASC; Strict control of analgesia with regional blocks or potent oral analgesics (avoid deep sedation); Immediate interruption of the procedure: at any sign of organic toxicity; Well-ventilated environment.

The BSA classification offers an objective model for clinical decision-making, reinforcing the safety of phenol procedures and allowing their responsible use according to risk.^90^

### Analgesia and Sedation

To minimize the risk of arrhythmias and allow monitoring for signs of intoxication, oral analgesia (avoiding medications that prolong the QT interval) and potent topical analgesia or regional blockade are recommended, rather than deep sedation. Sedation can mask important symptoms, such as changes in speech and alertness, that indicate intoxication. It is recommended that the procedure be stopped immediately if there is any evidence of organ toxicity.^57^, ^91^, ^92^

### Safety Breaks

Breaks of 10–15 minutes should be instituted after each 0.5% of BSA treated, allowing partial elimination of phenol between sessions and the return of the QTc interval to baseline.^57^, ^62^, ^90^

### Continuous Cardiac Monitoring

Multiparametric monitoring (ECG/BP, HR, and O_2_ saturation) should be performed for early detection of arrhythmias or other adverse cardiac events whenever the treated area is greater than 1.5% of BSA or the procedure time is longer than 30 minutes.^58^, ^91^, ^92^

### Intravenous Hydration

Intravenous hydration before and during the procedure helps minimize renal toxicity and facilitates the excretion of phenol through the urinary system, reducing its systemic concentration. This support can be provided in an outpatient setting, provided the office is adequately equipped and complies with current health regulations for intravenous fluid administration and emergency support. It is recommended to administer 500 mL of Ringer's Lactate before the procedure begins, adding 500 mL for each 1.5% of treated BSA, or 30 minutes into the procedure.^90^, ^91^ Intravenous hydration with Ringer's Lactate is preferred in procedures involving phenol due to its balanced composition, which contributes to electrolyte and acid-base stability during the procedure. Unlike saline solution, which contains only sodium and chloride in high concentrations, Ringer's Lactate contains additional electrolytes such as potassium and calcium—important for cardiac stability—and lactate, which is metabolized to bicarbonate, helping to prevent metabolic acidosis often associated with systemic phenol absorption. Furthermore, it has a lower risk of hypernatremia and hyperchloremic acidosis. Although there are no specific studies directly comparing these solutions to phenol peels, the choice of Ringer's Lactate reflects a well-established and physiologically sound clinical practice for maintaining homeostasis in situations of increased metabolic stress.^93^

### Environmental Control and Staff Protection

It is essential that the room be well-ventilated to dissipate phenol vapors, reducing the risk of toxic inhalation. Measures such as keeping windows open, keeping fans and/or air conditioning on, and positioning a portable fan near the patient's nostrils to divert vapors from the airway are highly recommended.^94^

Personal protective equipment is mandatory for all professionals involved, including a waterproof apron, thick nitrile gloves, an N95 mask, and protective eyewear.^94^

All materials used in the procedure must be disposed of in rigid containers for hazardous waste, ensuring safe collection and minimizing the risks of environmental exposure.^90^

### Formulations, Quality Control, and Safe Preparation

It is essential that the physician performing deep peels with phenol-croton oil prepare the formulation immediately before use, following scientifically validated formulas, such as those proposed by Hetter or Baker, with a known, standardized, and reproducible composition.^19^, ^84^ However, it is important to emphasize that the active ingredients are purchased from compounding pharmacies, whose quality certification, traceability, and compliance with current health regulations must be rigorously verified.

The current indiscriminate marketing of phenol and *Croton tiglium* oil, including online and to non-medical professionals, poses a serious health risk. Phenol must have a pharmaceutical-grade purity certification seal and be diluted in absolute alcohol (100%) in a controlled manner.^95^ Croton oil, in turn, has high phytochemical variability between different batches and suppliers. Studies using liquid chromatography, mass spectrometry, and HPTLC (High-Performance Thin Layer Chromatography) have shown that many commercial products do not contain the same profile of phorbol esters, compounds directly responsible for the depth of action and the inflammatory pattern of peels.^56^, ^96^, ^97^

Furthermore, stability analyses have shown that the oil composition changes with storage, resulting in the degradation of fatty acids and the possible loss of expected clinical action.^98^ Therefore, choosing reliable suppliers with up-to-date analytical reports and rigorous purity control is essential.

The formulation should be prepared in a screw-top glass bottle, mixed continuously to prevent separation of components, and applied immediately after preparation. For staff protection, appropriate PPE is required, including a waterproof apron, thick nitrile gloves, goggles, and an N95 mask. Handling should be done in a well-ventilated environment to minimize the risk of inhalation of toxic vapors.^90^

Regarding the emulsifier, scientific literature recommends the preferred use of Novisol®, due to its safety, compatibility with phenol, and role in emulsion stability, as demonstrated in stability studies of the Hetter formula.^99^, ^100^

Finally, to reduce the risk of systemic toxicity, it is recommended that the total volume of phenol used in a single session does not exceed 10 mL, regardless of the concentration used.^19^, ^84^

### Emergency Interventions

The presence of cardiopulmonary resuscitation equipment is mandatory for the management of serious adverse events, such as Acute Respiratory Distress Syndrome (ARDS) or cardiopulmonary arrest. Furthermore, it is essential that physicians performing phenol procedures be highly qualified and have up-to-date training in Advanced Cardiac Life Support (ACLS), ensuring adequate response capacity to critical complications.^76^, ^84^, ^90^

#### Cutaneous Risks

**Figure 9:**
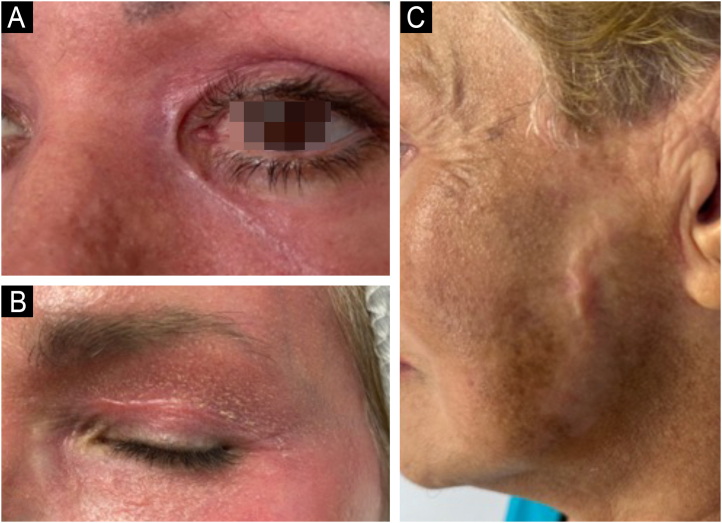
Hypertrophic scars after phenol-croton peeling in the eyelid region (A and B) and preauricular region (C). Photo: Dr. Felipe Ribeiro.

**Figure 10:**
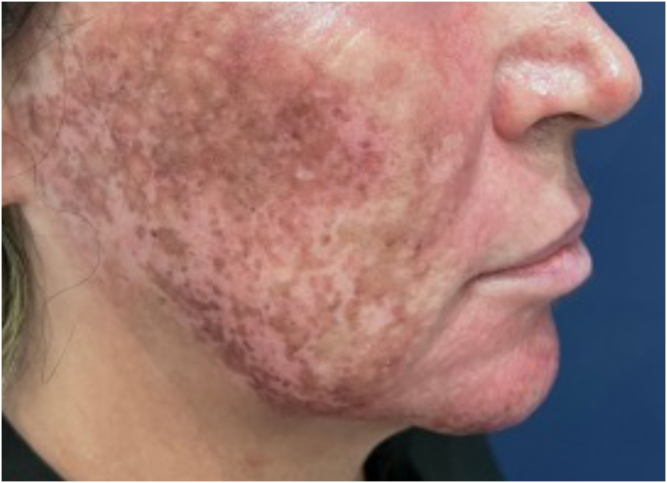
Abnormal healing one month after phenol-croton 35%/1.2% peeling in a patient with frontal fibrosing alopecia. Photo: Dr. Felipe Ribeiro.

In addition to the recognized systemic risks of phenol, the use of this agent in dermatology is also associated with significant local complications. These include infections, permanent scarring (Fig. 9), pigmentary changes, ocular damage, and impaired healing, particularly in patients with frontal fibrosing alopecia (FFA).^92^, ^101^ (Fig. 10).

FFA compromises skin regeneration due to the loss of follicular stem cells, which play a fundamental role in healing. Recent studies, such as that by Landau et al. (2024),^101^ highlight that patients with AFF have a significantly higher risk of scarring complications, making phenol-croton peeling a contraindication in these cases. These findings support the need for phenol procedures to be performed exclusively by trained physicians capable of assessing and managing individual risks. A detailed analysis of local complications would warrant a separate article, given its relevance and complexity.

## Conclusions

The use of phenol in dermatology, widely used in procedures such as chemical peels and onychocryptosis treatment, offers effective and unparalleled results. However, its use requires extreme caution due to the risk of systemic toxicity, such as cardiac arrhythmias and organ failure. This reinforces the need for rigorous protocols, especially when applying to larger areas such as the face, including cardiac monitoring, intravenous hydration, safety pauses, and segmented application, always in a controlled environment and under the supervision of specialist physicians.

Careful patient selection is essential to minimize local complications, such as impaired healing in specific conditions, such as FFA. Therefore, not only prior risk assessment but also expertise in conducting the procedure are of fundamental importance, making phenol procedures suitable only for physicians.

Additional studies are needed to determine safe doses and explore the long-term systemic effects of phenol, as well as reinforce the need for strict regulation and public awareness to ensure the safe, ethical, and responsible use of this substance. The lack of standardization in ready-made formulas and the widespread use by non-physicians pose serious risks to public safety.

## Financial support

None declared.

## Authors’ contributions

Carolina Reato Marçon: Design and planning of the study; collection, analysis, and interpretation of data; drafting and editing of the manuscript; critical review of the content; effective participation in research orientation.

## Conflicts of interest

None declared.

## Research data availability

The entire dataset supporting the results of this study was published in this article.

## Editor

Luciana P. Fernandes Abbade
